# Pulmonary embolism with patent Foramen Ovale: a distinct clinical entity demanding tailored management and screening

**DOI:** 10.3389/fmed.2025.1635735

**Published:** 2025-07-10

**Authors:** Mustafa Ajam, Ran Ran

**Affiliations:** ^1^Department of Cardiology, Oregon Health and Science University, Portland, OR, United States; ^2^Department of Emergency and Critical Care Medicine, Oregon Health and Science University, Portland, OR, United States

**Keywords:** pulmonary embolism, hypoxia, echocardiography, agitated saline, patent Foramen Ovale

## Introduction

Pulmonary embolism (PE) causes a wide range of clinical presentations. While computed tomography (CT) angiogram remains the primary diagnostic tool, consensus guidelines urge incorporating biomarkers and echocardiography to predict outcomes and define the treatment pathway (1). However, consensus is lacking on an important element of echocardiography in PE: the use of agitated saline. This technique is rarely performed but may offer unprecedented value, especially when hypoxemia is present. Herein, we describe the pathophysiology of hypoxemia in PE with an emphasis on right-to-left intracardiac shunting as an underrecognized but vital mechanism, the logistics and value of performing agitated saline exams, and the implications of positive shunt studies on management of hypoxemia as well as anticoagulation.

## Mechanisms of gas exchange abnormalities in pulmonary embolism

PE initiates a cascade of cardiopulmonary changes that can disrupt gas exchange. There is primarily an increase in alveolar dead space (2, 3), which can lead to hypercapnia, without necessarily causing hypoxia. In awake patients, hyperventilation compensates for CO_2_ buildup (4), but this is impaired in sedated or paralyzed individuals. Moreover, if cardiac output remains unchanged, blood flow to well-perfused lung areas increases, creating areas of low ventilation-perfusion ratio and potentially causing hypoxemia (5), especially when the central venous oxygen saturation is low (6). Hypoxemia worsens further when these regions suffer from poor ventilation, such as in the presence of concurrent pneumonia or alveolar hemorrhage (7). Fortunately, hypoxemia from these mechanisms is usually correctable with supplemental oxygen, as ventilation remains intact or elevated in many PE patients. However, this approach is not effective in the presence of a Patent Foramen Ovale (PFO) and right-to-left shunt. PFOs are common in the general population, and this high prevalence is mirrored in patients with PE (8), likely with higher incidence of right-to-left shunt due to increased right atrial pressure compared to the left (9). Therefore, utilizing available diagnostic tools to better understand the various factors influencing gas exchange in PE is essential, as it directly informs management strategies.

## Diagnostic utility of echocardiography in PE

CT angiography confirms the presence of a clot but does not indicate its age. A clot may have been present for days or weeks without symptoms, but the onset of another condition, like sepsis, can exacerbate the hemodynamic effects of PE. This underscores the complementary role of echocardiography, as it provides insight into the current hemodynamic status (10). If shock is present, echocardiograms help identify its type, moving beyond the common but overly simplistic term “obstructive shock.” Categorizing PE as either a low cardiac output or a normal/high output state provides a clearer picture of the patient's condition, allowing for more targeted treatment strategies (1). Echocardiography can also help differentiate the many aforementioned causes of hypoxemia by evaluating for right-to-left shunt, but only if paired with agitated saline.

When performing echocardiography to investigate the presence of a PFO, the current recommendation is to combine agitated saline with physiologic maneuvers that transiently raise right atrial pressure (11). For awake patients, timing the Valsalva maneuver release phase to coincide with contrast appearance in the right atrium provides a dynamic snapshot of the shunt. In sedated or ventilated patients, abdominal compression and inspiratory holds can be used similarly (12). While these maneuvers may sound straightforward to perform, they can be challenging in encephalopathic patients. Moreover, it is worth considering whether these additional physiologic maneuvers are truly necessary in hypoxemic patients. If the hypoxemia is caused by a right-to-left shunt, it should be detectable at rest, without the need to further increase right-sided pressures. In this context, the PE itself may already serve as the physiological maneuver, triggering the shunt without requiring additional interventions. This concern becomes even more significant if a clot-in-transit is detected, as these maneuvers should be avoided to reduce the risk of paradoxical embolism across the Foramen Ovale.

## Dynamic anatomy and physiology of PFO

**Figure 1:**
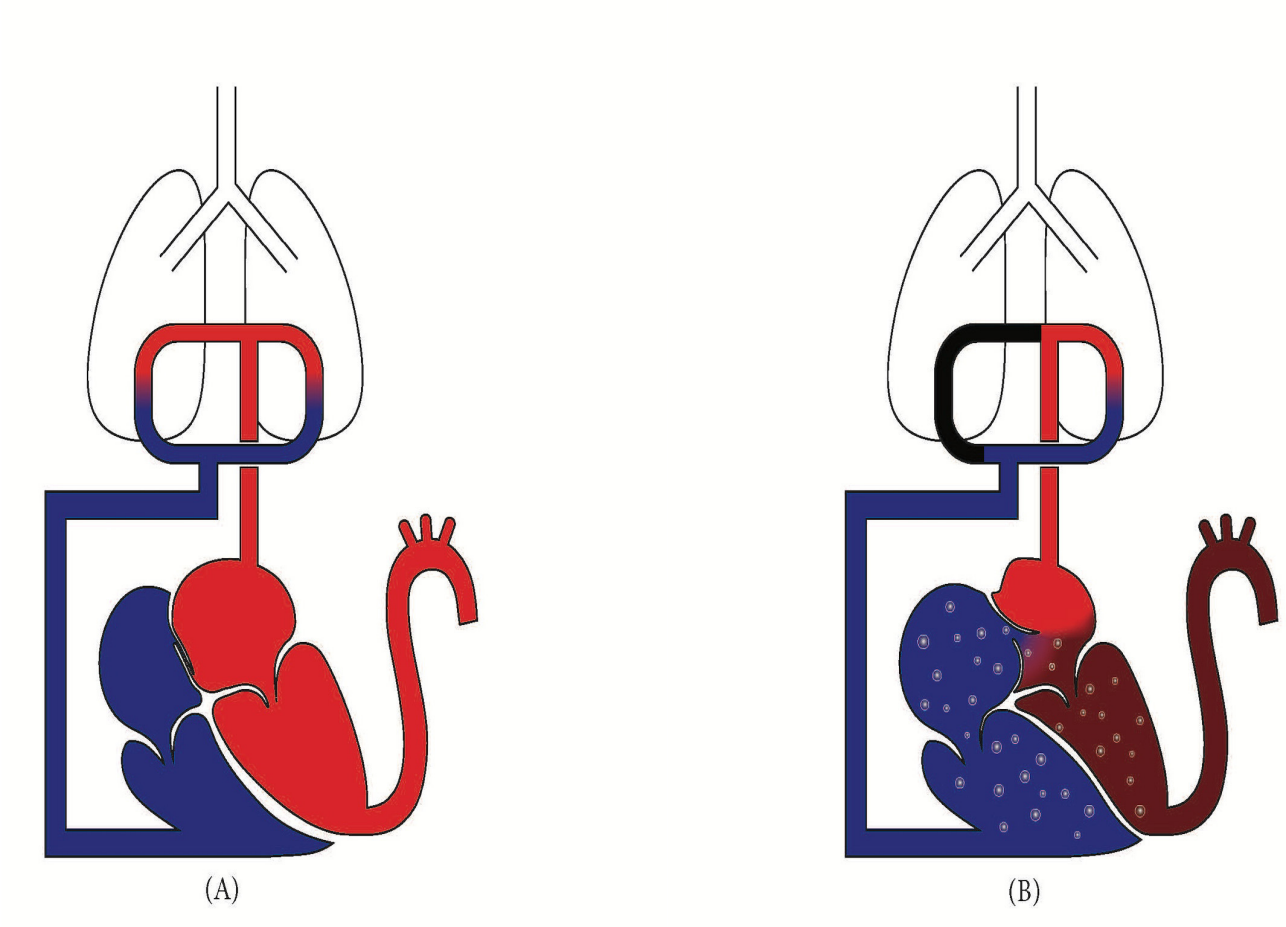
**(A)** Normal physiology with foramen Ovale closed. Higher left atrial pressure prevents right-to-left shunt. **(B)** In acute PE, right ventricular strain elevates right atrial pressure, reversing the pressure gradient and opening the foramen Ovale, resulting in a right-to-left shunt that bypasses the pulmonary circulation.

PFO, rather than being just a hole in the heart, is more accurately described as gaps between the septum primum and secundum. These separations, which can range from circular to elliptical or even tunneled, enable dynamic shunting, depending on the pressure gradient between the left and right atria (13). This gradient is crucial; a patient who had no signs of PFO on a previous echocardiogram may now display one after a PE, not because the PE eroded a hole, but because it increased right atrial pressure, reopening a path that had been closed, yet not sealed, since birth (Figure 1). Key echocardiographic findings such as elevated right ventricular systolic pressure or leftward shift of the interventricular septum, indicate the ideal conditions for right-to-left shunting. This is especially true when left-sided filling pressures are low, as evidenced by a hyperdynamic left ventricle in the absence of mitral stenosis or regurgitation (14). The extent of the resulting hypoxia is determined by the shunt fraction. If the left atrial blood is already at high saturation, situated on the flat portion of the oxygen-hemoglobin dissociation curve, noticeable hypoxia will only occur with a large shunt or low central venous oxygen saturation (15), which is commonly seen in low cardiac output states. This nuanced relationship between PFO size, pressure gradients, and oxygen levels offers a fresh perspective on hypoxia in PE, one that requires careful attention when evaluating these patients and planning their management.

## Clinical management and considerations in PE with PFO-related right-to-left shunt

Positive pressure ventilation such that provided by mechanical ventilators can be perilous in these patients. Sedation, which is commonly used alongside mechanical ventilation, can decrease systemic vascular resistance, leading to shifting of the interventricular septum and promoting right-to-left shunting. Furthermore, the increased pulmonary vascular resistance from positive pressure can exacerbate the shunt. Lastly, using positive pressure ventilation to correct refractory hypoxemia may be futile, especially when the underlying issue is primarily a perfusion defect rather than a ventilatory one. In such cases, strategies aimed at increasing systemic vascular resistance, alongside pulmonary vasodilators, may help restore balance between the right and left atrial pressures, thereby minimizing the shunt fraction (1).

Another key aspect of managing PE with PFO involves anticoagulation. While anticoagulation remains the first-line treatment for PE, its use in subsegmental PE is still debated (16). However, a positive agitated saline study heralds the potential for paradoxical embolisms, transforming small clots from annoyance to potentially lethal projectiles. This raises an important question: for patients with subsegmental PE who show signs of worsening right ventricular pressure load such as interval opening of Foramen Ovale, should anticoagulation be prioritized to reduce the risk of future complications and paradoxical embolism? Incorporating echocardiographic findings into the decision-making process could help guide more informed decisions regarding anticoagulation in this subset of patients. Future research is needed to clarify the best approach.

## Conclusion

In conclusion, TTE with agitated saline offers invaluable insights into the underlying mechanisms of hypoxia in pulmonary embolism. With the relatively high prevalence of PFO and its impact on both management and outcomes, agitated saline can provide the missing piece in many challenging cases, particularly when right-to-left shunting is demonstrated at rest, without the need for any physiologic maneuvers. While the current guidelines may not specify its use in PE, the evidence and physiology we have explored strongly suggests that it should be considered, especially in patients with hypoxia. This simple yet effective diagnostic tool can help us make more informed and precise decisions.

## References

[1] Konstantinides SV Meyer G Becattini C Bueno H Geersing G-J Harjola V-P. 2019 ESC guidelines for the diagnosis and management of acute pulmonary embolism developed in collaboration with the European Respiratory Society (ERS). Eur Respir J. (2019) 54:1901647. 10.1183/13993003.01647-2019 31473594

[2] Itti E Nguyen S Robin F Desarnaud S Rosso J Harf A. Distribution of ventilation/perfusion ratios in pulmonary embolism: an adjunct to the interpretation of ventilation/perfusion lung scans. J Nucl Med. (2002) 43:1596–602 12468507

[3] Robin ED Julian DG Travis DM Crump CH. A physiologic approach to the diagnosis of acute pulmonary embolism. N Engl J Med. (1959) 260:586–91. 10.1056/NEJM195903192601204 13632933

[4] Cvitanic O Marino PL. Improved use of arterial blood gas analysis in suspected pulmonary embolism. Chest. (1989) 95:48–51. 10.1378/chest.95.1.48 2491801

[5] Altemeier WA Robertson HT McKinney S Glenny RW. Pulmonary embolization causes hypoxemia by redistributing regional blood flow without changing ventilation. J Appl Physiol. (1985) 85:2337–43. 10.1152/jappl.1998.85.6.2337 9843561

[6] Tsang JY Lamm WJ Starr IR Hlastala MP. Spatial pattern of ventilation-perfusion mismatch following acute pulmonary thromboembolism in pigs. J Appl Physiol. (1985) 98:1862–8. 10.1152/japplphysiol.01018.2004 15591291

[7] Tsang JY Hogg JC. Gas exchange and pulmonary hypertension following acute pulmonary thromboembolism: has the emperor got some new clothes yet? Pulm Circ. (2014) 4:220–36. 10.1086/675985 25006441 PMC4070768

[8] Chiche O Castellani M Doyen D Moceri P Baudouy D Saady R. Prevalence of patent foramen ovale and stroke in pulmonary embolism patients. Eur Heart J. (2013) 34:P1142. 10.1093/eurheartj/eht308.P1142

[9] Konstantinides S Geibel A Kasper W Olschewski M Blümel L Just H. Patent foramen ovale is an important predictor of adverse outcome in patients with major pulmonary embolism. Circulation. (1998) 97:1946–51. 10.1161/01.CIR.97.19.1946 9609088

[10] Douglas PS Garcia MJ Haines DE Lai WW Manning WJ Patel AR. ACCF/ASE/AHA/ASNC/HFSA/HRS/SCAI/SCCM/SCCT/SCMR 2011 Appropriate use criteria for echocardiography. A report of the American college of cardiology foundation appropriate use criteria task force, American society of echocardiography, American heart association, American society of nuclear cardiology, heart failure society of America, heart rhythm society, society for cardiovascular angiography and interventions, society of critical care medicine, society of cardiovascular computed tomography, society for cardiovascular magnetic resonance American college of chest physicians. J Am Soc Echocardiogr. (2011) 24:229–67. 10.1016/j.echo.2010.12.008 21338862

[11] Mitchell C Rahko PS Blauwet LA Canaday B Finstuen JA Foster MC. Guidelines for performing a comprehensive transthoracic echocardiographic examination in adults: recommendations from the American society of echocardiography. J Am Soc Echocardiogr. (2019) 32:1–64. 10.1016/j.echo.2018.06.004 30282592

[12] Bernard S Churchill TW Namasivayam M Bertrand PB. Agitated saline contrast echocardiography in the identification of intra- and extracardiac shunts: connecting the dots. J Am Soc Echocardiogr. (2021) 34:1–12. 10.1016/j.echo.2020.09.013 34756394

[13] Hara H Virmani R Ladich E Mackey-Bojack S Titus J Reisman M. Patent foramen ovale: current pathology, pathophysiology, clinical status. JACC. (2005) 46:1768–76. 10.1016/j.jacc.2005.08.038 16256883

[14] Silvestry FE Cohen MS Armsby LB Burkule NJ Fleishman CE Hijazi ZM. Guidelines for the echocardiographic assessment of atrial septal defect and patent foramen ovale: from the American society of echocardiography and society for cardiac angiography and interventions. J Am Soc Echocardiogr. (2015) 28:910–58. 10.1016/j.echo.2015.05.015 26239900

[15] Hemodynamics. Practical Cardiovascular Medicine. (2022). p. 790–814. 10.1002/9781119832737.ch36

[16] Stevens SM Woller SC Baumann Kreuziger L Doerschug K Geersing G-J Klok FA. Antithrombotic therapy for VTE disease: compendium and review of CHEST guidelines 2012–2021. Chest. (2024) 166:388–404. 10.1016/j.chest.2024.03.003 38458430

